# Evaluation of *NTP42*, a novel thromboxane receptor antagonist, in a first-in-human phase I clinical trial

**DOI:** 10.3389/fphar.2023.1296188

**Published:** 2023-12-21

**Authors:** Helen M. Reid, Mark Maginn, C. Michael Perkins, Eamon P. Mulvaney, Malcolm Boyce, Takahiro Yamamoto, B. Therese Kinsella

**Affiliations:** ^1^ ATXA Therapeutics Limited, UCD Conway Institute of Biomolecular and Biomedical Research, University College Dublin, Belfield, Dublin, Ireland; ^2^ Hammersmith Medicines Research, London, United Kingdom

**Keywords:** thromboxane, safety, thromboxane receptor, *NTP42*, thromboxane receptor antagonist

## Abstract

**Background:** The thromboxane receptor (TP) antagonist *NTP42* is in clinical development for treatment of cardiopulmonary diseases, such as pulmonary arterial hypertension. In this randomized, placebo-controlled Phase I clinical trial, *NTP42*, administered as the oral formulation *NTP42:KVA4*, was evaluated for safety, tolerability, pharmacokinetics (PK), and pharmacodynamics (PD) in healthy males.

**Methods:** The first-in-human trial had three Parts: A, single ascending dose (SAD) study with seven groups given 0.25–243 mg *NTP42:KVA4* or placebo; B, food effect study where one SAD group (9 mg) was also given *NTP42:KVA4* or placebo after a high-fat breakfast; C, multiple ascending dose study with three groups given 15–135 mg *NTP42:KVA4* or placebo once-daily for 7 days.

**Results:** Seventy-nine volunteers participated. No serious adverse events occurred, where any drug- or placebo-related adverse events were mild to moderate, with no correlation to *NTP42:KVA4* dose. *NTP42* was rapidly absorbed, yielding dose proportional increases in exposure after single and repeat dosing. PK confirmed that, with a clearance (T_1/2_) of 18.7 h, *NTP42:KVA4* is suited to once-daily dosing, can be taken with or without food, and does not accumulate on repeat dosing. At doses ≥1 mg, *NTP42* led to complete and sustained inhibition of thromboxane-, but not ADP-, induced platelet aggregation *ex vivo*, with direct correlation between *NTP42* exposure and duration of PD effects.

**Conclusion:** Orally administered *NTP42:KVA4* was well tolerated, with favorable PK/PD profiles and evidence of specific TP target engagement. These findings support continued clinical development of *NTP42:KVA4* for cardiopulmonary or other relevant diseases with unmet needs.

**Clinical Trial Registration:** clinicaltrials.gov, identifier NCT04919863.

## Introduction

The prostanoid thromboxane (TX) A_2_ and the free-radical derived isoprostane 8-iso-prostaglandin (PG) F_2α_ signal through the thromboxane receptor (TP) to regulate multiple processes including platelet aggregation, constriction, and proliferation of vascular and pulmonary smooth muscle. They also mediate potent *pro*-inflammatory, *pro*-mitogenic, and *pro*-fibrotic effects within the lungs, heart, and wider cardiovascular system. Imbalances in the levels of TXA_2_, 8-iso-PGF_2α_, or the TP have been implicated in a range of cardiopulmonary diseases (Davi et al., 2012; Capra et al., 2013; Capra et al., 2014; Fontana et al., 2014; Al-Naamani et al., 2016; West et al., 2016; Badimon et al., 2021; Ashton, 2023), and aberrant TXA_2_/TP signaling contributes to cardiac dysfunction in multiple preclinical disease-models (Hoffmann et al., 1993; Nakamura et al., 1996; Francois et al., 2004; Wacker et al., 2006; Francois et al., 2008; Wacker et al., 2009; West et al., 2016; West et al., 2019).

*NTP42* is a novel TP antagonist in clinical development for the treatment of cardiopulmonary diseases such as pulmonary arterial hypertension (PAH) (Mulvaney et al., 2020a; Mulvaney et al., 2020b; Mulvaney et al., 2022). *NTP42* inhibits both TXA_2_-and 8-iso-PGF_2α_-induced signaling, being highly specific for the human TP, acting as neither a TP agonist, or agonist or antagonist of other prostanoid receptors (Mulvaney et al., 2020a).

PAH is a rare disease characterized by structural and functional changes in the pulmonary vasculature resulting in elevated pulmonary vascular resistance (PVR), right ventricular (RV) hypertrophy and, ultimately, RV failure and death (Vonk Noordegraaf and Galie, 2011; Naeije and Manes, 2014; Ryan and Archer, 2014). Preclinical efficacy data shows that *NTP42* can attenuate key PAH disease hallmarks, many of which are also found in related cardiopulmonary conditions (Mulvaney et al., 2020a; Mulvaney et al., 2020b; Mulvaney et al., 2022). In the monocrotaline (MCT)-and Sugen/Hypoxia-induced PAH models, the active pharmaceutical ingredient (API) *NTP42* attenuated pulmonary pathologies and consequent RV effects (Mulvaney et al., 2020a; Mulvaney et al., 2020b). Moreover, *NTP42:KVA4*, an oral formulation of *NTP42* developed as an investigational medicinal product (IMP) for clinical use, was evaluated in both the MCT-PAH and pulmonary artery banding (PAB) model of RV overload (Mulvaney et al., 2022). In the MCT-PAH model, efficacy of *NTP42:KVA4* was consistent with previous findings using the *NTP42* API. In the PAB model, *NTP42:KVA4* promoted a beneficial pattern of cardiac hypertrophy, resulting in significantly improved cardiac function. Moreover, findings in the PAB model pointed to a direct cardioprotective effect for *NTP42:KVA4* as a component of its clinical potential, not only in PAH, but also in more prevalent cardiac conditions involving RV dysfunction.

In a Phase II clinical trial in PAH patients that evaluated Terbogrel, which also targets the TXA_2_/TP pathway, the development of acute leg pain in participants led to the study being prematurely terminated during enrolment (Langleben et al., 2002). Notably, Terbogrel acts as both a TP antagonist and TXA_2_ synthase (TXS) inhibitor (Langleben et al., 2002). As subsequently reported, leg pain occurred due to Terbogrel’s inhibition of TXS which, while reducing TXA_2_ generation, resulted in a shift towards synthesis of prostacyclin, a potent pain inducer (Berkenkopf and Weichman, 1988; Shindo et al., 1991; Murata et al., 1997; Langleben et al., 2002; Popp et al., 2009). In contrast to Terbogrel, *NTP42* is a highly selective TP antagonist and does not act as a TXS inhibitor (Mulvaney et al., 2020a). Thus, *NTP42* will not lead to increased prostacyclin synthesis or to pain induction associated with dual TP antagonists and TXS inhibitors, such as Terbogrel (Kinsella and Reid, 2018; Kinsella and Reid, 2019; Mulvaney et al., 2020a).

The objective of this study was to evaluate the safety, tolerability, pharmacokinetic (PK) and pharmacodynamic (PD) properties of the selective TP antagonist *NTP42*, delivered orally as *NTP42:KVA4*, in healthy adult volunteers.

## Material and methods

### Study design and subjects

This first-in-human Phase I, single-center, double-blinded, randomized, placebo-controlled trial aimed to assess the safety and tolerability of *NTP42:KVA4* in healthy male volunteers. It also aimed to evaluate the PK profile and PD effect of *NTP42*, after single and repeated doses of *NTP42:KVA4*.

Eligible subjects were aged 18–55 years, with a body mass index of 18–30 kg/m^2^, and with no clinically significant medical conditions. All subjects provided written informed consent. The trial was conducted in 3 parts: Part A, a single ascending dose (SAD) study; Part B, a food effect study, and Part C, a multiple ascending dose (MAD) study. In Part A, fifty-five fasted subjects were randomly assigned to receive a single oral dose of placebo or *NTP42:KVA4* (0.25–243 mg). The 0.25 mg starting dose was selected based on a review of preclinical toxicokinetic data and predicted to have no pharmacological activity. For Part B, one group from Part A (9 mg *NTP42:KVA4*) received a second dose of *NTP42:KVA4* or placebo after consuming a high fat breakfast. In Part C, twenty-four fasted subjects were randomly assigned to three increasing dose Groups to receive repeat, once daily oral *NTP42:KVA4* (15–135 mg) or matching placebo for seven consecutive days.

At an initial screening visit, prospective trial participants were assessed for eligibility, including a full physical exam. Those eligible with no clinically significant electrocardiogram (ECG), vital signs or laboratory test (hematology, coagulation, biochemistry, and urinalysis) findings were admitted to the clinical site and randomized on Day −1 for all parts and dose groups. As the Phase I trial was conducted during the height of the Coronavirus 2 (Covid) pandemic (24th May 2021 through 13th January 2022), specific screening and other safety measures were introduced, both at admission and throughout all aspects of the trial.

For each dose group in Part A, a sentinel cohort of two subjects received either *NTP42:KVA4* or placebo (1:1 randomization) before the remaining subjects were dosed. Eligible subjects were confined to the clinical site from the day before dosing (Day −1), administered a single dose of *NTP42:KVA4* or placebo on Day 1, and discharged from the clinic on the morning of Day 3. Subjects returned to the clinical site for a follow-up review at 7–10 days after their final dose for end-of-study safety assessments (including physical examination, hematology, coagulation, biochemistry, urinalysis, and ECG). For Groups 4, 5, 6, and 7 (9, 27, 81, and 243 mg *NTP42:KVA4* doses), PK plasma collection and PD assessment was also performed at the follow-up visit.

Part C subjects were confined to the clinical site for the duration of the in-house period from Day −1, administered once daily oral doses of *NTP42:KVA4* or placebo from Day 1 to Day 7, discharged from the clinic on the morning of Day 9, and returned to the clinic 7–10 days after their last dose for a follow-up review and end-of-study safety assessments (including physical examination, hematology, coagulation, biochemistry, urinalysis, ECG, PK plasma collection and PD assessment).

The study was conducted by Hammersmith Medicines Research (London, United Kingdom) in compliance with Good Clinical Practice (GCP), which has its origins in the Declaration of Helsinki. A Safety Review Group (SRG) was established to determine and approve the dose levels for all parts of the trial. The SRG reviewed blinded safety, tolerability, and anonymized PK and PD data for each previous dose group before proceeding with the next dose escalation or before progressing from Part A to Parts B or C.

### Study drug

*NTP42* (API; N-(tert-butylcarbamoyl)-5-cyano-2-((4'-(trifluoromethoxy) -[1,1′-biphenyl]-3-yl)oxy)benzenesulfonamide) is formulated with the excipient Kollidon^®^ VA 64 at a ratio of 1:4 (drug substance: polymer) for oral administration where the IMP is referred to as *NTP42:KVA4*. The matching placebo for *NTP42:KVA4* was prepared as a fill of the corresponding weight of the excipient Kollidon^®^ VA 64 only. *NTP42:KVA4* and its matching placebo were manufactured and dispensed in accordance with current good manufacturing practices (cGMP) with the required doses given as an oral suspension (*NTP42:KVA4*) or in solution (placebo) in water. *NTP42:KVA4* and the placebo were given to subjects in a blinded manner.

### Safety and tolerability

Safety assessments, including physical examination, vital signs, 12-lead ECG, laboratory safety tests (hematology, coagulation, clinical chemistry, and urinalysis), for all parts of the study were performed at screening, pre-dose, following dosing up to 48 h after the last dose, and at follow-up.

### Pharmacokinetics

In Parts A and B, PK parameters for *NTP42* were assessed following collection of serial blood samples at pre-dose and frequently up to 48 h after each dose and at follow-up. In Part C, samples were taken before and frequently up to 18 h after the first dose (Day 1); before and 12 h after the second dose (Day 2); before each dose on Days 3–6; before and frequently up to 48 h after the last dose and at follow-up. PK analyses were performed at Analytical Services International (London, United Kingdom) using liquid chromatography/tandem mass spectrometry (LC-MS/MS) and a validated method for the quantification of *NTP42* in human plasma. Noncompartmental PK analyses following the linear trapezoidal linear interpolation calculation method were performed using Phoenix WinNonlin (Version 8.3).

### Statistical methods

This study was an exploratory trial and there were no null hypotheses to be tested. All descriptive statistical analyses were performed using Statistical Analysis Software (SAS; Version 9.4).

### Pharmacodynamics

In Part A, blood was taken for assessment of TXA_2_ (U46619)- or, as controls, vehicle- and ADP-induced platelet aggregation before and frequently up to 48 h after dosing on Day 1 and at follow-up. In Part C, samples were taken before and frequently up to 12 h after the first dose (Day 1); before each dose on Days 2–6; before and frequently up to 48 h after the last dose (Day 7), and at follow-up. There was no PD assessment undertaken for Part B.

Blood was collected using 3.2% sodium citrate monovettes and centrifuged at 150 *g* for 15 min at room temperature to prepare platelet rich plasma (PRP). Platelet poor plasma (PPP) was obtained by re-centrifugation of the blood samples at 2,500 × *g* for 20 min at room temperature. A platelet count was performed and the PRP adjusted to 250 × 10^9^ platelets/L by diluting PRP with autologous PPP.

Platelet aggregation assays were performed on the Helena AggRAM aggregometer, using the internally validated method developed prior to Trial commencement. In these assays, PRP samples were maintained at 37 °C and stirred throughout analyses. PRP samples from each individual were treated with either the TP agonist U46619 (1.5 µM in duplicate for all dose groups) or, as controls, saline or adenosine diphosphate (10 µM ADP; an agonist for the purinergic P2Y_1_/P2Y_12_ receptors expressed in platelets). Platelet aggregation was monitored for 6 min following stimulation.

## Results

### Subject disposition

A total of 79 subjects were enrolled, 55 in the SAD study (Part A) and 24 in the MAD study (Part C). In Part B, 8 subjects who had received a single dose of IMP or placebo in Part A received a second dose after consuming a high-fat breakfast. While no subjects withdrew from the trial, six subjects from Part A were not available to participate in Part B as originally intended. Therefore, to ensure a cross-over design, six replacements were recruited. While the mean age (39 years) of the subjects participating in Part B, including these replacements, was higher than in Part A (32 years), the range of 22–51 years in Part B was in line with that of all other groups. Subject demographics and key baseline parameters (*e.g*., vital signs and clinical laboratory data) are summarized in Table 1 and in Supplementary Table S1.

**TABLE 1:** Summary of demographic details of trial participants.

Variable			*Part A* ^a^ ^,^ ^b^		*Part B* ^b^		*Part C* ^a^ ^,^ ^c^	
			Placebo (fasted) (N = 16)	*NTP42:KVA4* ^d^ 0.25–243 mg (fasted) (N = 39)	Placebo (fed) (N = 2)	*NTP42:KVA4* ^d^ 9 mg (fed) (N = 6)	Placebo QD (N = 6)	*NTP42:KVA4* ^d^ 15–135 mg QD (fasted) (N = 18)
Age (Years; Mean ± SD)			31.9 ± 8.8	32.0 ± 10.6	32.5 ± 14.9	41.2 ± 8.0	32.8 ± 9.1	36.2 ± 10.4
Gender (Male; n)			16	39	2	6	6	18
Race	White (n)		15	31	2	5	5	14
	Asian (n)		1	3	0	0	0	0
	Black or African American (n)		0	3	0	0	1	3
	Other (n)		0	2	0	1	0	1
Height (cm; Mean ± SD)			178.5 ± 7.7	178.0 ± 7.5	180.0 ± 2.8	170.9 ± 7.4	177.5 ± 5.3	177.9 ± 8.4
Weight (kg; Mean ± SD)			78.47 ± 14.1	77.09 ± 12.0	90.80 ± 6.2	76.03 ± 16.1	80.50 ± 10.0	78.91 ± 14.1
BMI (kg/m^2^; Mean ± SD)			24.46 ± 2.9	24.07 ± 3.0	28.0 ± 1.0	23.50 ± 3.0	25.52 ± 2.7	24.83 ± 2.5
Systolic BP^e^ (mmHg; Mean ± SD)		Supine	109.6 ± 6.38	112.6 ± 6.65	108.0 ± 4.24	111.8 ± 6.74	114.7 ± 6.53	113.4 ± 9.82
		Standing	103.3 ± 10.3	102.2 ± 11.2	96.0 ± 15. 6	98.0 ± 6.69	108.3 ± 8.19	105.2 ± 11.2
Diastolic BP^e^ (mmHg; Mean ± SD)		Supine	67.31 ± 7.54	67.77 ± 7.05	68.0 ± 8.49	72.0 ± 5.93	71.5 ± 5.61	68.4 ± 8.15
		Standing	71.0 ± 10.0	66.67 ± 12.0	61.50 ± 7.78	68.3 ± 8.64	65.2 ± 10.9	69.8 ± 11.5
Heart Rate^e^ (bpm; Mean ± SD)		Supine	58.69 ± 11.8	61.87 ± 10.4	60.0 ± 4.24	53.7 ± 7.31	55.5 ± 7.31	61.8 ± 12.4
		Standing	84.38 ± 20.1	88.26 ± 15.6	102.0 ± 28.3	73.8 ± 15.7	80.7 ± 10.0	85.1 ± 15.6
QTcF Interval^f^ (msec; Mean ± SD)			390.0 ± 10.68	391.1 ± 16.1	386.8 ± 0.24	398.6 ± 15.5	397.2 ± 16.6	398.2 ± 18.5

Abbreviations: BMI, body mass index; BP, blood pressure; bpm, beats per minute; N, total number of subjects; n, number of applicable subjects; mmHg, millimeters of mercury; msec, millisecond; QD, *quaque die* or once daily; QTcF, QT, interval corrected according to Fridericia’s formula; SD, standard deviation.

^a^ All subjects in Part A and Part C were given *NTP42:KVA4* or Placebo under fasted conditions.

^b^ Overall mean (range) age, weight, height, and BMI, for subjects in Parts A and B were 32.0 years (18–54 years), 78.33 kg (55.3–104.9 kg), 178.2 cm (163–197 cm), and 24.59 kg/m2 (19.7–29.9 kg/m2), respectively.

^c^ For Part C, the mean (range) age, weight, height, and BMI, were 35.3 years (20–55 years), 79.19 kg (64.4–112.3 kg), 177.8 cm (165–195 cm), and 24.97 kg/m2 (21.4–29.5 kg/m2), respectively.

^d^ *NTP42:KVA4* delivered as an oral suspension in water.

^e^ For all subjects, supine blood pressure and heart rate were measured after the subject had been resting for 10 min, followed by standing blood pressure and heart rate after 2 min in the standing position. Vital signs given in Table represent measurements taken pre-dose on Day 1 and are presented as Mean ± SD.

^f^ For all subjects, 12-lead electrocardiogram (ECG) measurements were taken in triplicate with subjects in a supine position. QTc, value was corrected for heart rate using Fridericia’s formula for correction.

### Safety and tolerability

All subjects completed the trial with no serious adverse events (SAEs), other significant adverse events (AEs) or AEs leading to subject withdrawal. As summarized in Table 2, all AEs observed after *NTP42:KVA4* or placebo dosing (*i.e*., treatment-emergent AEs, TEAEs) were classed as mild or moderate in severity.

**TABLE 2:** Summary of possible drug- and placebo-treatment-emergent adverse events.

	Number of subjects with ≥1 TEAE [total number of TEAEs]					
	*Part A* ^a^		*Part B*		*Part C* ^a^	
	Placebo (fasted) (N = 16)	*NTP42:KVA4* ^b^ 0.25–243 mg (fasted) (N = 39)	Placebo (fed) (N = 2)	*NTP42:KVA4* ^b^ 9 mg (fed) (N = 6)	Placebo QD (N = 6)	*NTP42:KVA4* ^b^ 15–135 mg QD (fasted) (N = 18)
Any TEAE^c^	4 [6]	11 [16]	0	0	3 [3]	5 [5]
Any drug- or placebo related TEAE^d^	3 [5]	6 [9]	0	0	0	2 [2]
Any SAE	0	0	0	0	0	0
Any TEAE leading to subject withdrawal	0	0	0	0	0	0
Any TEAE with mild as worst severity	3	6	0	0	3	3
Any TEAE with moderate as worst severity	1	5	0	0	0	2
Any TEAE with severe as worst severity	0	0	0	0	0	0
Any TEAE with life-threatening as worst severity	0	0	0	0	0	0

Abbreviations: N, total number of subjects; QD, *quaque die* or once daily; SAE, serious adverse event; TEAE, treatment emergent adverse event.

^a^ All subjects in Part A and Part C were given *NTP42:KVA4* or Placebo under fasted conditions.

^b^ *NTP42:KVA4* delivered as an oral suspension in water.

^c^ A TEAE, is defined as an adverse event (AE) occurring after a *NTP42:KVA4* or Placebo dose is given that was not present prior to dosing or an event that worsens in intensity or in frequency after dosing.

^d^ Drug- or placebo related AEs, were determined by the Principal Investigator to be related to either the study drug or placebo.

In Part A, TEAEs determined to be possibly related to the drug or placebo were reported following 0.25, 1, 3, and 27 mg *NTP42:KVA4* and placebo, but not 9, 81, or 243 mg *NTP42:KVA4* (Table 3). The most frequently observed drug- or placebo-related TEAE was dizziness, followed by orthostatic hypotension (Table 3). There was no correlation between *NTP42:KVA4* dose and overall TEAE incidence or intensity. In Part B, no TEAEs were reported after dosing of *NTP42:KVA4* or placebo in the fed state (Tables 2, 3). In Part C, while TEAEs were reported for placebo and 15, 45, and 135 mg *NTP42:KVA4*, of these, only 2 subjects dosed with 45 mg *NTP42:KVA4* had a TEAE considered as possibly *NTP42*-related (Tables 2, 4).

**TABLE 3:** Likely drug- and placebo-related treatment-emergent adverse events in parts a and B.

	Placebo^a^ (fasted) (N = 16)	Placebo (fed) (N = 2)	*NTP42:KVA4*									All subjects ** (N = 55)
			0.25 mg (fasted) (N = 2)	1 mg (fasted) (N = 4)	3 mg (fasted) (N = 4)		9 mg (fasted) (N = 11)	9 mg (fed) (N = 6)	27 mg (fasted) (N = 6)	81 mg (fasted) (N = 6)	243 mg (fasted) (N = 6)	
	Number of subjects with ≥1 TEAE [total number of TEAEs]											
Total^#^	3	–	1	1		2	–	–	2	–	–	9
Dizziness	2 [2]	–	1 [1]	–		1 [1]	–	–	–	–	–	4 [4]
Dizziness postural	–	–	–	–		1 [1]	–	–	1 [1]	–	–	2 [2]
Headache	–	–	–	–		1 [1]	–	–	–	–	–	1 [1]
Orthostatic hypotension	2 [2]	–	–	–		–	–	–	1 [1]	–	–	3 [3]
Orthostatic heart rate response increased	–	–	1 [1]	1 [1]		–	–	–	–	–	–	2 [2]
Nausea	1 [1]	–	–	–		–	–	–	–	–	–	1 [1]
Decreased appetite	–	–	–	–		–	–	–	1 [1]	–	–	1 [1]

Abbreviations: N, total number of subjects; TEAE, treatment emergent adverse event.

^a^ All placebo (Fasted) subjects in Part A. **All subjects in Parts A and B.

^#^Total number of subjects who experienced one or more TEAEs.

**TABLE 4:** Likely drug- and placebo-related treatment-emergent adverse events in part C.

	Placebo^a^ QD (N = 6)	*NTP42:KVA4* 15 mg QD (N = 6)	*NTP42:KVA4* 45 mg QD (N = 6)	*NTP42:KVA4* 135 mg QD (N = 6)	All subjects** (N = 24)
	Number (%) of subjects with ≥1 TEAE [total number of TEAEs]				
Total^#^	–	–	2	–	2
Headache	–	–	2 [2]	–	2 [2]

Abbreviations: N, total number of subjects; QD, *quaque die* or once daily; TEAE, treatment emergent adverse event.

^a^ All placebo (Fasted) subjects in Part C. **All subjects in Part C.

^#^Total number of subjects who experienced one or more TEAEs.

For Parts A-C, there were no clinically relevant changes in physical examination findings, laboratory parameters (Supplementary Table S2), or ECG. Vital signs were within normal limits or, if outside reference ranges, not deemed clinically significant (Supplementary Table S3). Minor, non-significant decreases in systolic blood pressure on standing were the most frequent observation in the *NTP42:KVA4-*or placebo-dose groups, followed by an increase in heart rate on standing.

### Pharmacokinetics

**FIGURE 1:**
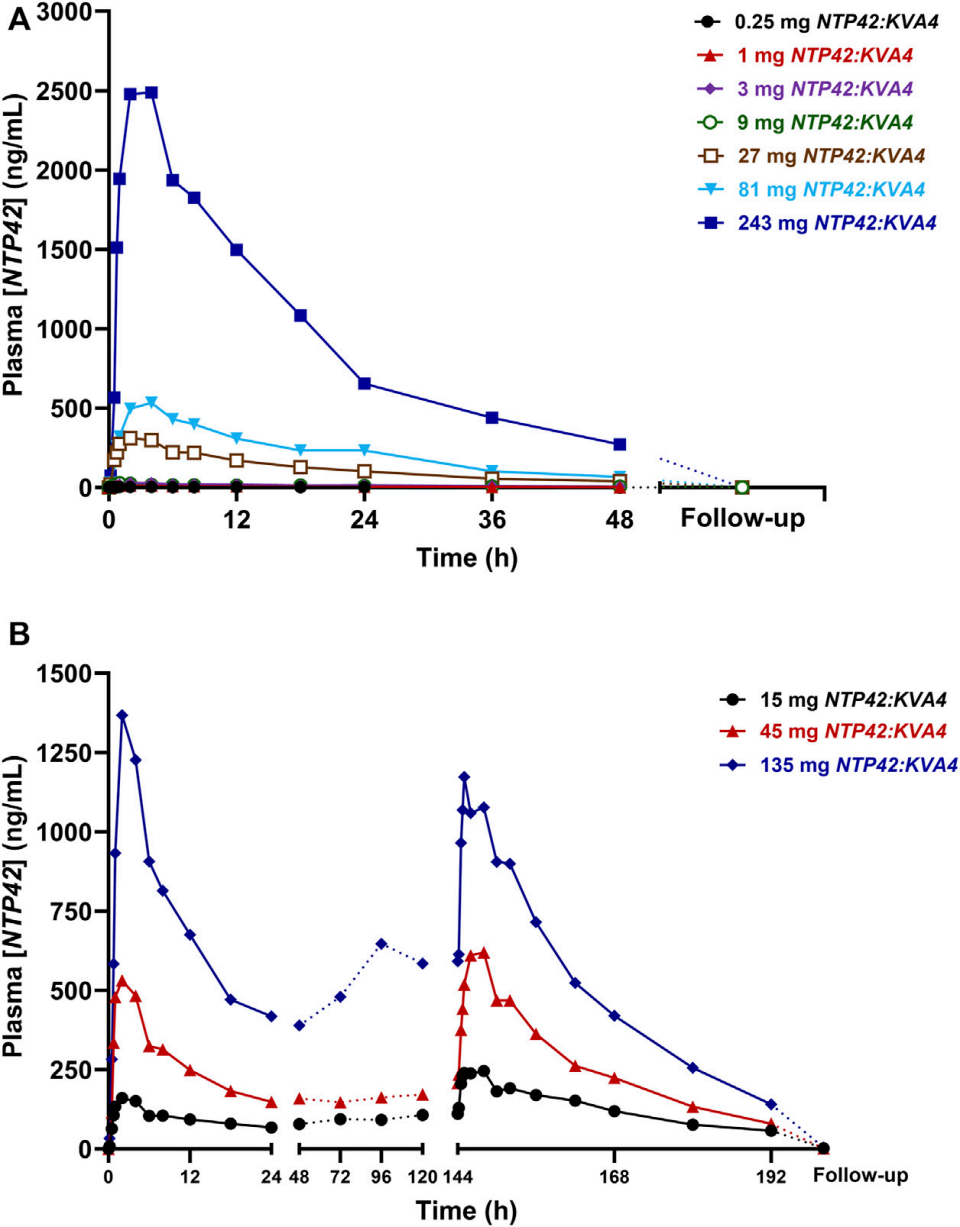
Mean *NTP42* Plasma Concentrations Following Single and Repeat Oral Doses of *NTP42:KVA4* in Fasting Healthy Male Subjects (Parts A and C) **(A,B)** Serial blood samples were collected pre-dose, frequently up to 48 h after the last dose and at follow-up (some 7–10 days after last *NTP42:KVA4* dose) from subjects given either single oral doses of placebo or 0.25 mg–243 mg *NTP42:KVA4* (Panel A) or repeat single daily oral doses of placebo or 15 mg–135 mg *NTP42:KVA4* for 7 days (Panel B). Data is presented as mean changes in concentrations of *NTP42* in plasma (Plasma [*NTP42*], ng/mL) from pre-dose to follow-up (some 7–10 days after the last *NTP42:KVA4* dose) as determined by liquid-chromatography/tandem mass spectrometry (LC-MS/MS) and a validated method for the quantification of *NTP42* in human plasma. The limit for quantitation (LoQ) for the bioanalytical assay was 1 ng/mL.

In Part A, *NTP42* was detected in plasma from all subjects receiving *NTP42:KVA4* (Figure 1A; Supplementary Figure S1A). *NTP42* was rapidly absorbed, being first detected 30 min or 10 min after single doses of 0.25 mg or ≥1 mg, respectively, with mean peak concentrations observed 2–4 h post-dose (C_max_; Table 5). Generally, mean C_max_ values increased proportionally with dose, *e.g.,* with 27-fold dose increase (9–243 mg), C_max_ increased 28-fold. Clearance of *NTP42* was similar across all dose groups showing a gradual decline over 24–48 h with terminal elimination (T_1/2_) from 15 to 23 h following doses ≥1 mg *NTP42:KVA4*. Total *NTP42* exposure (area under the curve, AUC) increased less than dose proportionally with *NTP42:KVA4* dose between 1 and 243 mg (156-fold following a 243-fold dose increase; Table 5).

**TABLE 5:** Summary of *NTP42* plasma pharmacokinetics following single doses of *NTP42:KVA4* in fasting healthy male subjects (parts A and B).

*NTP42 PK Parameter*		*NTP42:KVA4*							
		0.25 mg (fasted) (N = 2)	1 mg (fasted) (N = 4)	3 mg (fasted) (N = 4)	9 mg (fasted) (N = 11)	9 mg (fed) (N = 6)	27 mg (fasted) (N = 6)	81 mg (fasted) (N = 6)	243 mg (fasted) (N = 6)
C_max_ (ng/mL)	Geo Mean	2.74	12.5	27.7	87.5	64.5	325	528	2,459
	95% CI	(0.19; 39.6)	(9.19; 17.1)	(18.0; 42.7)	(73.2; 105)	(49.2; 84.6)	(226; 468)	(360; 774)	(1,488; 4,065)
T_max_ (h)	Median	2.5	2.0	3.1	2.0	7.0	2.0	3.0	2.0
AUC_inf_ (h∙ng/mL)	Geo Mean	134	323	823	1,801	1,818	6,695	11,705	50,279
	95% CI	(0.74; 24,151)	(244; 429)	(426; 1,588)	(1,367; 2,373)	(943; 3,505)	(5,124; 8,749)	(8,729; 15,696)	(31,426; 80,444)
T_½_ (h)	Mean	43.2	21.8	23.1	17.6	22.1	17.3	15.4	17.0
	SD	16.0	3.2	5.7	8.4	13.1	6.1	5.5	6.9

Abbreviations: AUC_inf_, Area under curve *versus* time curve from start of dose administration to infinity; CI: confidence interval; C_max_, Peak plasma concentration measured after dosing; Geo, Geometric; N, total number of subjects; PK, pharmacokinetic; SD, standard deviation; T_½_, Apparent terminal elimination half-life.; t_max_, Time to reach C_max_ or peak plasma concentration after dosing.

In Part B subjects, after consuming a high fat breakfast, absorption was slightly delayed with peak *NTP42* plasma concentrations observed 4–8 h after *NTP42:KVA4* dosing and the C_max_ decreased (87.5 ng/mL in fasted subjects, 64.5 ng/mL in fed state, Tables 5, 6). However, there was no overall difference in the AUC in the fed-compared to the fasted-states (Table 5; geometric mean AUC_inf_ after 9 mg *NTP42:KVA4,* 1,801 and 1,818 h∙ng/mL in fasted and fed states, respectively). ANOVA analysis confirmed that while food reduced C_max_, it had no effect on the exposure of *NTP42* (AUC_inf_; Table 6).

**TABLE 6:** Effect of food on *NTP42* plasma pharmacokinetic parameters after 9 mg *NTP42:KVA4* given in fed and fasted states.

Parameter	Geometric LS means		Fed vs*.* fasted	
	*NTP42:KVA4* 9 mg (fed) (N = 6)	*NTP42:KVA4* 9 mg (fasted) (N = 6)	Geometric mean ratio	90% CI
C_max_ (ng/mL)	64.5	87.8	0.73	0.62, 0.87
AUC_inf_ (h∙ng/mL)	1,818.3	1,810.1	1.00	0.90, 1.12

Abbreviations: AUC_inf_, Area under curve *versus* time curve from start of dose administration to infinity; CI: confidence interval; C_max_, Peak plasma concentration measured after dosing; LS, Least-squares; N, total number of subjects.

In Part C, *NTP42* was detected in plasma from all subjects receiving repeated doses of *NTP42:KVA4* (15–135 mg), being first detected at 10 min after initial dosing (Figure 1B; Supplementary Figure S1B). Peak *NTP42* concentrations were observed at 2 h post-dose after the first dose (Day 1) and at 4 h post-dose on Day 7 (Table 7). C_max_ increased close to dose proportionally on Day 1, while on Day 7, following repeat dosing, C_max_ increased less than dose proportionally (4.5-fold increase in C_max_ with 9-fold dose increase). Mean T_1/2_ values ranged from 14.9 to 26.1 h on Day 1 and 19.6–23.1 h on Day 7 (Table 7). Total exposure (AUC) after single and repeat dosing increased less than dose proportionally in Part C, *i.e.*, AUC_τ_ increased 7.0-fold on Day 1 and 4.24-fold on Day 7 with a 9-fold dose increase.

**TABLE 7:** Summary of *NTP42* plasma pharmacokinetics following repeat doses of *NTP42:KVA4* in fasting healthy male subjects (part C).

*NTP42* PK parameter		*NTP42:KVA4*					
		15 mg QD (N = 6)		45 mg QD (N = 6)		135 mg QD (N = 6)	
		Day 1	Day 7	Day 1	Day 7	Day 1	Day 7
C_max_ (ng/mL)	Geo mean	160	249	524	640	1,293	1,124
	95% CI	(113; 228)	(166; 372)	(423; 650)	(447; 915)	(739; 2,262)	(671; 1,883)
T_max_ (h)	Median	2.0	3.0	2.0	3.0	2.0	1.1
AUC_inf_ (h∙ng/mL)	Geo mean	–	7,239	–	13,887	–	26,787
	95% CI	–	(3,964; 13,218)	–	(9,018; 21,385)	–	(16,953; 42,328)
AUC_τ_ (h∙ng/mL)	Geo mean	2,249	3,868	6,414	8,667	15,676	16,407
	95% CI	(1,632; 3,099)	(2,420; 6,182)	(5,138; 8,007)	(6,011; 12,496)	(9,911; 24,793)	(10,449; 25,762)
T_½_ (h)	Mean	26.1	23.1	14.9	19.6	16.6	21.5
	SD	8.3	6.3	2.9	3.8	6.8	4.7
R_ac(AUC)_	Mean Ratio	–	1.75	–	1.38	–	1.15
	95% CI	–	(1.41, 2.08)	–	(1.06, 1.70)	–	(0.626, 1.67)
R_ac(Cmax)_	Mean Ratio	–	1.57	–	1.25	–	1.00
	95% CI	–	(1.27, 1.88)	–	(0.942, 1.55)	–	(0.449, 1.56)

Abbreviations: AUC_inf_, Area under curve *versus* time curve from start of dose administration to infinity; AUC_τ_: Area under curve across a dosing interval (0–24 h); CI: confidence interval; C_max_, Peak plasma concentration measured after dosing; Geo, Geometric; N, total number of subjects; PK, pharmacokinetic; QD, *quaque die* or once daily; R_ac(AUC)_, Accumulation ratio for AUC (calculated from AUC_t_, at steady state and AUC_t_, after single dose); R_ac(Cmax)_: Accumulation ratio for C_max_ (calculated from C_max_ at steady state and C_max_ after single dose); SD, standard deviation; T_½_, Apparent terminal elimination half-life.; t_max_, Time to reach C_max_ or peak plasma concentration after dosing.

In all Part C subjects, *NTP42* was quantifiable pre-dose on Days 2–7 with mean C_trough_ indicating that steady-state exposure was attained between Days 3–6 (Figure 1B). Moreover, mean Day 1–7 accumulation ratios of *NTP42* for AUC_τ_ and C_max_ were higher for the lowest dose tested (15 mg) and decreased as doses increased (Table 7). Mean ratios for AUC_τ_ and C_max_ of 1.15 and 1.00, respectively, in subjects receiving repeated dosing of 135 mg *NTP42:KVA4* suggests little to no accumulation of *NTP42* at the highest dose level.

### Pharmacodynamics

As a PD read-out and surrogate marker of clinical efficacy for TP antagonists (Guth et al., 2004; Gaussem et al., 2005; Sakariassen et al., 2009; Fiessinger et al., 2010; Lesault et al., 2011), the ability of *NTP42* to inhibit thromboxane (TX)A_2_- but not adenosine diphosphate (ADP)-induced platelet aggregation *ex vivo* was assessed after single and repeat dosing of *NTP42:KVA4*. As food consumption is known to interfere with *ex vivo* platelet assays, aggregations were not performed for Part B.

**FIGURE 2:**
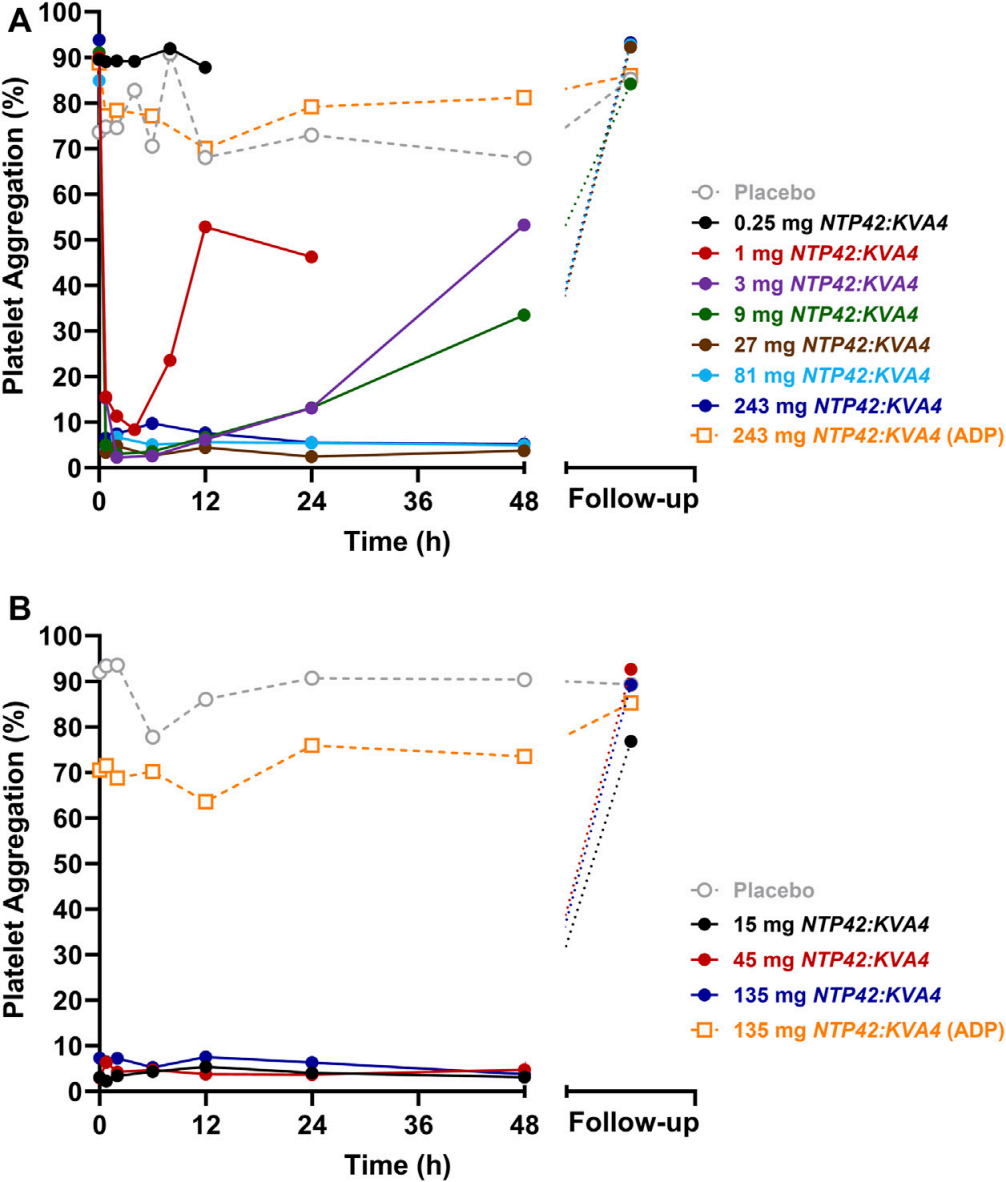
Effect of Single and Repeat Oral Dosing of *NTP42:KVA4* on Human Platelet Aggregation Following U46619 and ADP Stimulation **(A)** Aggregation of platelets obtained from subjects given single oral doses of placebo (grey dashed line) or 0.25 mg–243 mg *NTP42:KVA4* (solid-colored lines) following stimulation with 1.5 µM U46619 or 10 µM ADP (243 mg dose group only; orange dashed line). **(B)** Aggregation of platelets obtained from subjects given repeat oral doses of placebo (grey dashed line) or 15 mg–135 mg *NTP42:KVA4* (solid-colored lines) following stimulation with 1.5 µM U46619 or 10 µM ADP (135 mg dose group only; orange dashed line). In both panels, data are presented as mean changes in maximal platelet aggregation (Platelet Aggregation; %) from pre-dose to follow-up (some 7–10 days after the last *NTP42:KVA4* dose) as determined using the Helena AggRAM aggregometer.

No platelet aggregation was induced by the drug vehicle (saline), either at baseline (pre-dose) and after single or repeat dosing of *NTP42:KVA4* at all doses tested (data not shown). At pre-dose, both platelet agonists, U46619 and ADP, induced strong platelet aggregation responses in all subjects (Figure 2; Supplementary Figure S2).

After single oral dosing at 0.25 mg *NTP42:KVA4*, no inhibition of U46619-induced platelet aggregation was observed, consistent with the lack of a pharmacological effect of *NTP42* at this low dose (Figure 2A; black line). Following single dosing of ≥1 mg *NTP42:KVA4*, dose-dependent inhibition of U46619-induced platelet aggregation was observed with complete inhibition (≥90%) following ≥3 mg *NTP42:KVA4* that was sustained for up to 24 h (Figure 2A; Supplementary Figure S2A). Aggregation responses returned to baseline in a dose- and time-dependent manner, with all *NTP42:KVA4* doses (1–243 mg) back at pre-dose baseline levels at the follow-up assessment (7–10 days after dosing). No inhibition of U46619-induced platelet aggregation was observed following placebo (Figure 2A; dashed grey line).

In Part C, complete inhibition of U46619-induced platelet aggregation occurred at all doses tested (Figure 2B; Supplementary Figure S2B), which was sustained from 45 min after the first dose on Day 1 until at least 48 h after the last dose on Day 7. Aggregation responses returned to baseline at follow-up for most individuals with only 2 subjects in the 15 mg dose group showing 18% and 50% inhibition of aggregation remaining at this visit. There were no underlying characteristics in these two subjects compared to other subjects in the same group or, indeed, in those dosed with 45 mg or 135 mg *NTP42:KVA4* that might explain the more prolonged inhibition of U46619-induced platelet aggregation.

In both Parts A and C, a slight non-significant decrease (≤10%) in mean ADP-induced platelet aggregation from baseline was observed after single and repeat dosing at all *NTP42:KVA4* or placebo doses (Figure 2; orange dashed lines). The greatest decreases (≥20%) in ADP-induced responses were observed at 12 h post-dose in all subjects receiving *NTP42:KVA4*. However, a similar decrease in ADP-induced aggregation was also observed in subjects receiving the placebo. Overall, there was no substantive inhibition of ADP-induced platelet aggregation after single or repeat oral *NTP42:KVA4* dosing, confirming the specificity of *NTP42* for TXA_2_/TP-, and not ADP/(P2Y_1_/P2Y_12_)-, receptor responses.

## Discussion

This study is the first clinical evaluation of *NTP42*, a novel TP antagonist in development as a treatment for cardiopulmonary diseases such as PAH and other related conditions. *NTP42*, administered as the IMP *NTP42:KVA4,* was found to be safe and well-tolerated after single oral dosing up to 243 mg and repeat once-daily oral dosing up to 135 mg.

In all parts of the trial, there were no significant AEs or SAEs that either stopped dose escalation or resulted in subject withdrawal. Any drug- or placebo-related TEAEs were considered mild or moderate in severity, with no SAEs and no overall correlation in incidence or severity of the TEAE to *NTP42:KVA4* dose. Also, there was no correlation between TEAEs, either incidence or severity, with age, ethnicity, or other clinical characteristics of trial participants. The most common drug- or placebo-related TEAEs were dizziness, orthostatic hypotension, and headache. It was notable that in Part B, following food consumption, no TEAEs were observed in any subject. Thus, it is suggested that the TEAEs observed in Parts A and C may be procedural as subjects were fasted and supine for long periods before orthostatic assessments. Overall, there were no clinically significant changes in vital signs, ECGs, or safety laboratory tests at any of doses tested, including no changes in coagulation parameters such as activated partial thromboplastin time (aPTT) and international normalized ratio (INR).

*NTP42* was rapidly absorbed after single and repeat oral dosing of *NTP42:KVA4*, with dose proportional increases in exposure and favorable clearance rates indicating that *NTP42:KVA4* may be suited for use as a once-a-day oral medication. The data also suggests that *NTP42:KVA4* may be taken with or without food, another key benefit for patient compliance. Moreover, there was excellent correlation between *NTP42* plasma levels and PD effect. At doses ≥1 mg *NTP42:KVA4*, complete and sustained inhibition of U46619-, but not ADP-, induced aggregation was observed, confirming selective TP-target engagement. The duration of inhibition of U46619-induced aggregation was dependent on the plasma levels of *NTP42* after dosing, where U46619-induced platelet responses returned to baseline (pre-dose) levels in a dose-dependent manner.

Inhibition of TP-mediated platelet aggregation has been used to determine doses of TP antagonists to be tested for efficacy in early-stage clinical trials in multiple disease indications (Guth et al., 2004; Gaussem et al., 2005; Sakariassen et al., 2009; Fiessinger et al., 2010; Lesault et al., 2011; Richardson et al., 2013). While inhibition of TP-mediated platelet aggregation is not a regulatory-approved marker of efficacy in PAH patients *per se*, there is ample evidence for PAH being, in part, a prothrombotic state caused by the dysregulation of platelet activation, coagulation, fibrinolysis, and endothelial cell interaction (Rabinovitch, 2012). For example, Zhu et al. outlined a key role for platelet-derived transforming growth factor (TGF)-β in the metabolic reprogramming of pulmonary arterial smooth muscle cells and subsequent pulmonary vascular remodeling (Zhu et al., 2022). In that study, therapies that reduce platelet TGF-β release have strong potential to translate into clinically efficacious treatments for PAH (Zhu et al., 2022). Notably, Sotatercept, in clinical development for PAH, acts as a ligand trap for members of the TGF-β superfamily (Yung et al., 2020; Humbert et al., 2021; Hoeper et al., 2023). In Phase III trials, benefits for Sotatercept were seen across key clinical parameters, including the 6-min walk distance (6MWD) test, the primary clinical endpoint (Hoeper et al., 2023). However, using a multicomponent improvement (MCI) index, a composite measure based on pre-specified improvements in functional class (FC), 6MWD and NT-proBNP, only 40% of patients responded to Sotatercept delivered in combination with background PAH therapy (Hoeper et al., 2023). This latter finding demonstrates that there remains a clear need for novel therapies for PAH, in particular those with the potential to address all the clinical hallmarks of the disease, to lessen premature mortality while robustly improving patient quality-of-life.

Notwithstanding the potential benefits of TP antagonism acting through platelet-derived mechanisms in cardiopulmonary diseases such as PAH, or in other conditions, extensive data from preclinical models has shown *NTP42*’s efficacy in treating diverse pulmonary and cardiac pathologies (Mulvaney et al., 2020a; Mulvaney et al., 2020b; Mulvaney et al., 2022). With benefits in alleviating excessive vasoconstriction, pulmonary artery remodeling, as well as pulmonary inflammation and fibrosis in the preclinical setting, *NTP42* is anticipated to lead to a reduction in PVR, the regulatory-approved marker of hemodynamic efficacy in PAH. Moreover, our preclinical efficacy data using the PAB model demonstrates that *NTP42* may have a direct cardioprotective benefit, highly distinct from other PAH standard-of-care (SoC) therapies or indeed pipeline compounds (Mulvaney et al., 2022). These findings are relevant when considering that, in up to a third of patients with reduced PVR on PAH SoC therapy, RV function continues to decrease, with poorer survival outcomes (van de Veerdonk et al., 2011). Thus, the ideal PAH therapy should not only target pulmonary vasoconstriction and vascular remodeling but should also demonstrate direct cardioprotective effects and promote beneficial adaptation and maintenance or improvement in RV function.

Phase I clinical trials, by their nature, being typically conducted at a single clinical site, may suffer from a lack of ethnic diversity in the volunteer cohort recruited. As this FIH Phase I trial was conducted in healthy male, mostly white, Caucasian volunteers, it is acknowledged that the safety and tolerability findings and PK/PD profiles reported herein may differ in other diverse ethnic groups, females, and/or diseased patient cohorts. The safety, tolerability, PK/PD, and clinical efficacy profiles of *NTP42* will be investigated in Phase II/III trials in relevant patient cohorts.

In conclusion, this study showed that *NTP42:KVA4* is safe and well tolerated, with favorable PK/PD characteristics and clear evidence of specific TP target engagement. These findings, combined with compelling preclinical efficacy data (Mulvaney et al., 2020a; Mulvaney et al., 2020b; Mulvaney et al., 2022), support the continued development of *NTP42:KVA4* for the treatment of cardiopulmonary diseases, such as PAH, or other conditions involving aberrant TXA_2_/TP signaling.

## Data Availability

The datasets presented in this article are not readily available because trial data may only be made available under the terms of a Confidential Disclosure Agreement (CDA). Requests to access the datasets should be directed to therese.kinsella@atxatherapeutics.com.
