# Supplementary material for: Evaluation of NTP42, a novel thromboxane receptor antagonist, in a first-in-human phase I clinical trial
Source: Front Pharmacol. 2023 Dec 21;14:1296188. doi: 10.3389/fphar.2023.1296188 (PMC10764490; doi:10.3389/fphar.2023.1296188)
Supplement: Supplementary file 1 [file DataSheet1.PDF]

SUPPLEMENTAL MATERIAL

SUPPLEMENTAL TABLES

Supplemental Table 1. Summary of Key Clinical Laboratory Parameters at Baseline in Healthy Male Subjects.

Clinical Pathology Parameter[3]		***Parts A & B***[1]		***Part C***[1]	
		**Placebo (Fasted) (N=16)**	***NTP42:KVA4***[2] **0.25 – 243 mg (Fasted) (N=39)**	**Placebo QD (N=6)**	***NTP42:KVA4***[2] **15 – 135 mg QD (Fasted) (N=18)**
**Coagulation**	Activated Partial Thromboplastin Time (aPTT; sec)	31.11 ± 2.03	31.07 ± 2.25	31.7 ± 3.68	31.3 ± 2.63
	Prothrombin International Normalized Ratio (INR; Ratio)	0.936 ± 0.072	0.961 ± 0.061	0.960 ± 0.043	0.996 ± 0.101
**Haematology**	Leukocytes ($x10^9$ cells/L)	5.50 ± 1.04	5.31 ± 1.68	6.07 ± 0.86	5.14 ± 1.11
	Erythrocytes ($x10^{12}$ cells/L)	4.77 ± 0.33	4.88 ± 0.34	4.93 ± 0.23	4.84 ± 0.41
	Platelets ($x10^9$ platelets/L)	227.1 ± 25.7	219.9 ± 40.9	219 ± 54.0	223 ± 41.3
**Clinical Chemistry**	Alanine Aminotransferase (ALT; IU/L)	22.31 ± 5.97	23.795 ± 10.1	23.2 ± 8.61	25.5 ± 8.16
	Bilirubin (µmol/L)	16.8 ± 7.08	15.7 ± 7.17	15.9 ± 3.86	15.9 ± 5.62

Abbreviations: ALT, alanine aminotransferase; aPTT, activated partial thromboplastin time; INR, prothrombin international normalized ratio; IU, international units; N, total number of subjects; µmol, micromoles; QD, *quaque die* or once daily; SD, standard deviation; sec, second.

[1] Subjects in Part A and Part C were given *NTP42:KVA4* or Placebo under fasted conditions. In Part B, a group of subjects in Part A (9 mg *NTP42:KVA4* dose group) were given a second dose under fed conditions.

[2] *NTP42:KVA4* delivered as an oral suspension in water.

[3] For all subjects, blood was taken for clinical pathology assessments on the day before the first dose (Day -1), where the Table provides data from key parameters as Mean ± SD.

Supplemental Table 2. Summary of Key Clinical Laboratory Parameters following Single & Repeat Doses of *NTP42:KVA4* in Healthy Male Subjects.

**Clinical Pathology Parameter**[3]		***Part A***[1]		***Part B***[1]		***Part C***[1]	
		**Placebo (Fasted) (N=16)**	***NTP42:KVA4***[2] **0.25 – 243 mg (Fasted) (N=39)**	**Placebo (Fed) (N=2)**	***NTP42:KVA4***[2] **9 mg (Fed) (N=6)**	**Placebo QD (N=6)**	***NTP42:KVA4***[2] **15 – 135 mg QD (Fasted) (N=18)**
**Coagulation**	Activated Partial Thromboplastin Time (aPTT; sec)	30.02 ± 3.36	30.43 ± 3.30	25.0 ± 3.54	29.9 ± 2.99	31.3 ± 2.77	30.4 ± 2.24
	Prothrombin International Normalized Ratio (INR; Ratio)	0.959 ± 0.077	0.980 ± 0.043	0.930 ± 0.057	0.948 ± 0.053	0.982 ± 0.038	1.021 ± 0.87
**Haematology**	Leukocytes (x10^9 cells/L)	5.92 ± 1.01	5.34 ± 1.37	6.60 ± 0	4.72 ± 0.64	5.80 ± 0.53	5.24 ± 1.03
	Erythrocytes (x10^{12} cells/L)	4.85 ± 0.36	4.91 ± 0.33	4.4 ± 0	4.72 ± 0.34	4.82 ± 0.35	4.74 ± 0.35
	Platelets (x10^9 platelets/L)	226.7 ± 24.6	219.0 ± 35.9	247.0 ± 0	208.3 ± 38.6	228.0 ± 51.4	230.4 ± 52.4
**Clinical Chemistry**	Alanine Aminotransferase (ALT; IU/L)	21.8 ± 4.99	22.7 ± 9.54	26.0 ± 2.83	59.7 ± 12.40	22.2 ± 7.19	21.5 ± 7.24
	Bilirubin (µmol/L)	18.5 ± 9.43	17.5 ± 7.71	14.5 ± 1.91	17.6 ± 5.91	12.9 ± 4.23	13.6 ± 3.38

Abbreviations: ALT, alanine aminotransferase; aPTT, activated partial thromboplastin time; INR, prothrombin international normalized ratio; IU, international units; N, total number of subjects; µmol, micromoles; QD, *quaque die* or once daily; SD, standard deviation; sec, second.

[1] Subjects in Part A and Part C were given *NTP42:KVA4* or Placebo under fasted conditions. In Part B, a group of subjects in Part A (9 mg *NTP42:KVA4* dose group) were given a second dose under fed conditions.

[2] *NTP42:KVA4* delivered as an oral suspension in water.

[3] Blood was taken for clinical pathology assessments 24 hours after dosing in Part A and after the second dose in Part B. IN Part C, blood was taken for clinical pathology assessments 24 hours after the last dose, *i.e.*, Day 8. The Table provides data from key parameters as Mean ± SD.

Supplemental Table 3. Summary of Vital Signs (Blood Pressure & Heart Rate) Following Dosing in Healthy Male Subjects.

Variable		***Part A***[1]		***Part B***		***Part C***[1]	
		**Placebo (Fasted) (N=16)**	***NTP42:KVA4***[2] **0.25 – 243 mg (Fasted) (N=39)**	**Placebo (Fed) (N=2)**	***NTP42:KVA4***[2] **9 mg (Fed) (N=6)**	**Placebo QD (N=6)**	***NTP42:KVA4***[2] **15 – 135 mg QD (Fasted) (N=18)**
**Systolic BP**[3] (mmHg; Mean ± SD)	Supine	112.3 ± 7.29	111.2 ± 9.29	108.0 ± 8.49	109.7 ± 5.68	112.2 ± 6.49	108.9 ± 8.00
	Standing	99.7 ± 9.65	100.6 ± 11.6	92.5 ± 7.78	98.3 ± 11.9	104.8 ± 12.0	101.7 ± 9.07
**Diastolic BP**[3] (mmHg; Mean ± SD)	Supine	68.5 ± 7.94	67.0 ± 7.94	72.5 ± 10.6	72.3 ± 6.71	72.8 ± 6.49	67.0 ± 7.88
	Standing	67.9 ± 12.6	66.2 ± 14.6	68.5 ± 0.71	68.7 ± 9.00	71.3 ± 8.24	68.4 ± 7.94
**Heart Rate**[3] (bpm; Mean ± SD)	Supine	61.3 ± 11.0	62.3 ± 10.6	67.5 ± 0.71	53.2 ± 10.6	56.7 ± 10.6	58.6 ± 9.42
	Standing	91.0 ± 22.1	90.3 ± 20.9	109 ± 45.25	78.0 ± 18.7	83.5 ± 14.6	84.9 ± 16.6

Abbreviations: BP, blood pressure; bpm, beats per minute; N, total number of subjects; mmHg, millimeters of mercury; QD, *quaque die* or once daily; SD, standard deviation.

[1]All subjects in Part A and Part C were given *NTP42:KVA4* or Placebo under fasted conditions.

[2] *NTP42:KVA4* delivered as an oral suspension in water.

[3] For all subjects, supine blood pressure and heart rate were measured after the subject had been resting for 10 min, followed by standing blood pressure and heart rate after 2 min in the standing position. Vital signs given in Table represent measurements taken 4 hours after dosing on Day 1 and are presented as Mean ± SD.

SUPPLEMENTAL FIGURE LEGENDS

Supplemental Figure 1

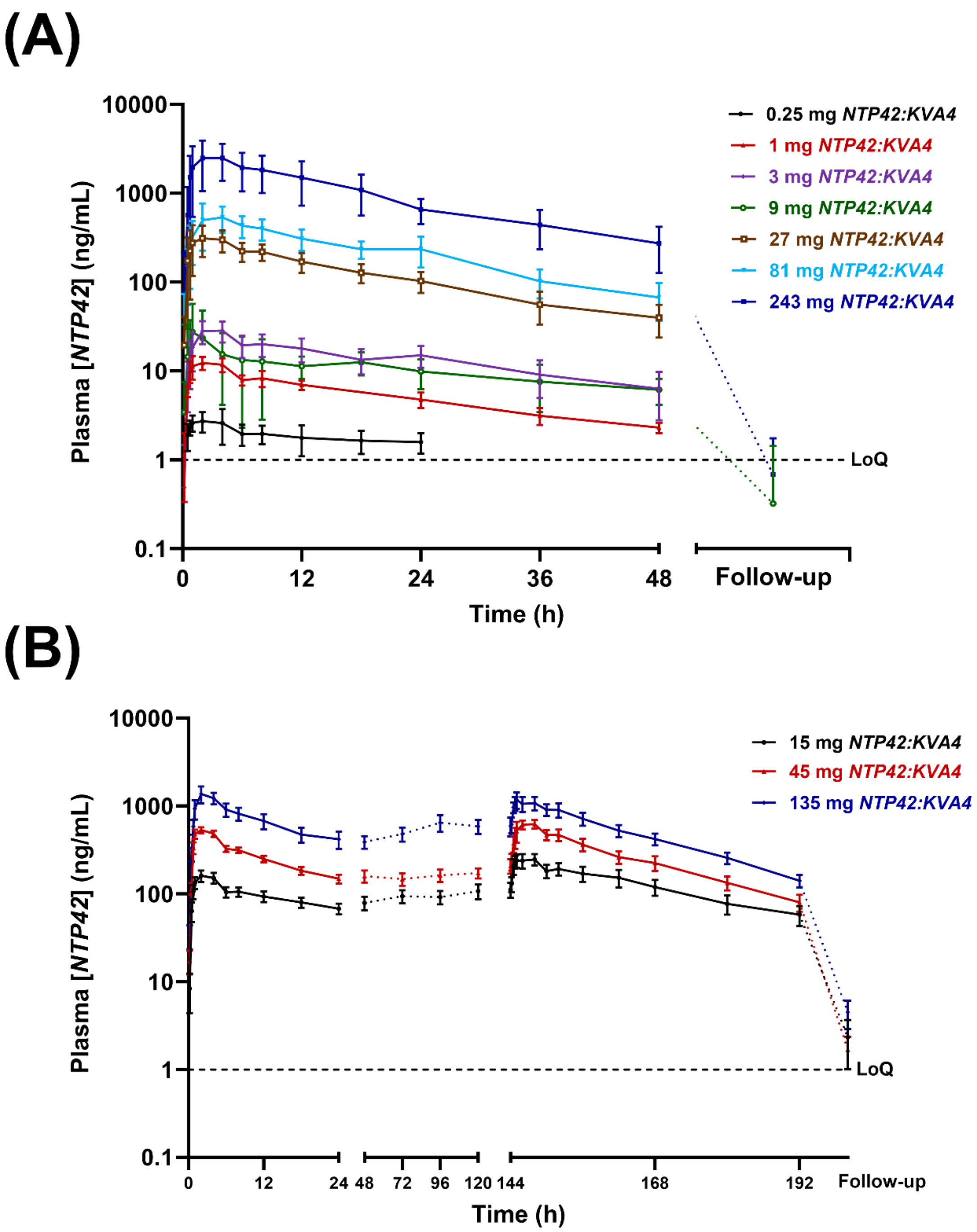

Supplemental Figure 1: Mean *NTP42* Plasma Concentrations Following Single and Repeat Oral Doses of *NTP42:KVA4* in Fasting Healthy Male Subjects (Parts A & C)

Panels A & B: Changes in plasma concentrations of *NTP42* (Plasma [*NTP42*], ng/mL; Mean ± SEM) at pre-dose, up to 48 h after single (Panel A) or repeated (Panel B) dosing, and at follow-up some 7 – 10 days after the last *NTP42:KVA4* dose are presented on a semi-log plot. The limit for quantitation (LoQ) for the bioanalytical assay was 1 ng/mL and is shown as a dashed line on the charts in Panels A & B.

Supplemental Figure 2

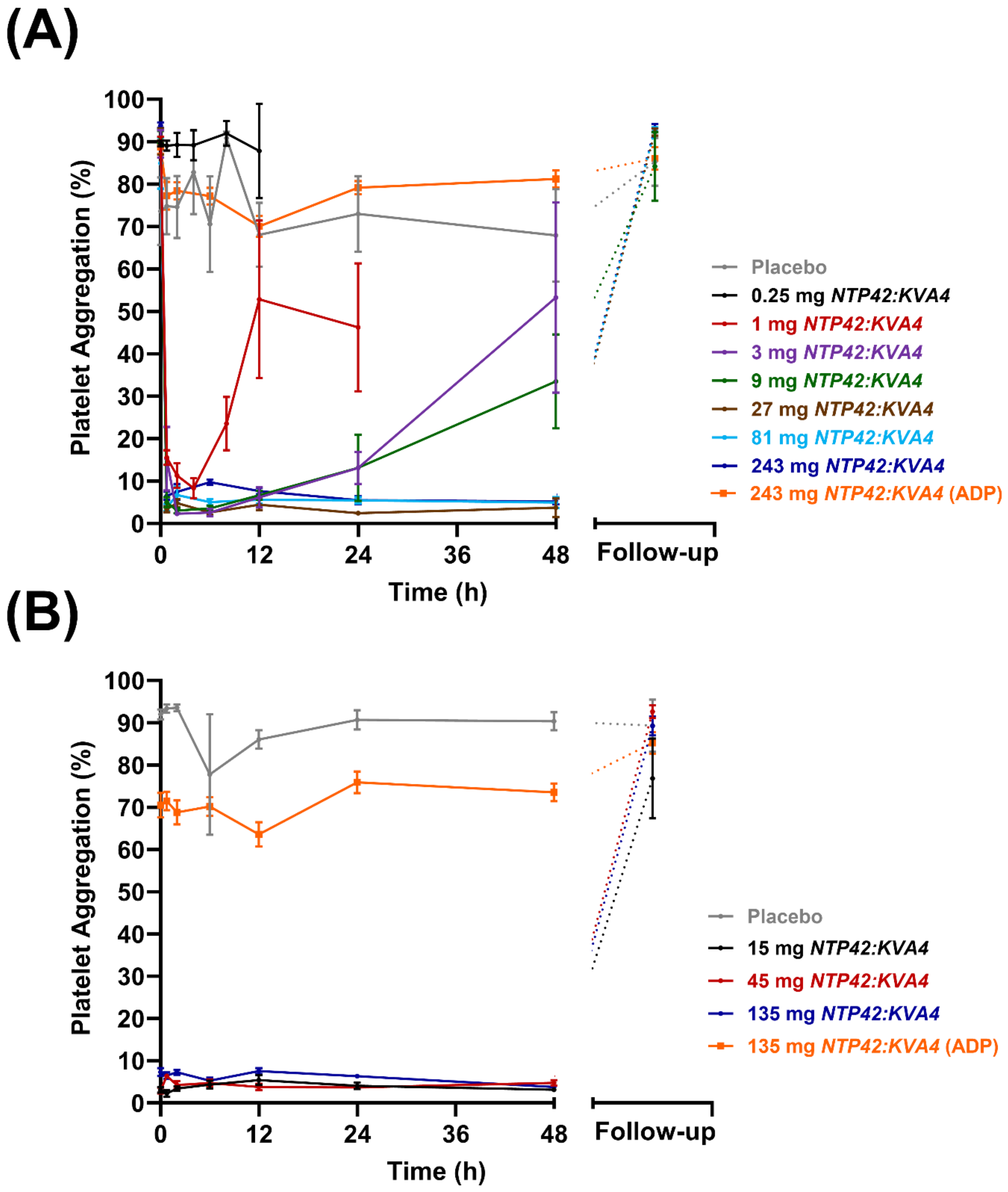

Supplemental Figure 2: Effect of Single and Repeat Oral Dosing of *NTP42:KVA4* on Human Platelet Aggregation Following U46619 & ADP Stimulation

Panels A & B: Changes in maximal platelet aggregation (Platelet Aggregation, %; Mean ± SEM) at pre-dose, up to 48 h after single (Panel A) or repeated (Panel B) dosing, and at follow-up some 7 – 10 days after the last *NTP42:KVA4* dose as determined using the Helena AggRAM aggregometer.
